# External validation, radiological evaluation, and development of deep learning automatic lung segmentation in contrast-enhanced chest CT

**DOI:** 10.1007/s00330-023-10235-9

**Published:** 2023-09-29

**Authors:** Krit Dwivedi, Michael Sharkey, Samer Alabed, Curtis P. Langlotz, Andy J. Swift, Christian Bluethgen

**Affiliations:** 1 Department of Infection, Immunity & Cardiovascular Disease, Medical School, University of Sheffield, Sheffield, UK; 2 Academic Department of Radiology, Royal Hallamshire Hospital, Glossop Road, Sheffield, S10 2JF USA; 3 3DLab, Sheffield Teaching Hospitals NHS Trust, Sheffield, UK; 4 Stanford Center for Artificial Intelligence in Medicine and Imaging (AIMI), Stanford University, Sheffield, USA

**Keywords:** Tomography, X-ray computed, Deep learning, Lung, Hypertension, pulmonary

## Abstract

**Objectives:**

There is a need for CT pulmonary angiography (CTPA) lung segmentation models. Clinical translation requires radiological evaluation of model outputs, understanding of limitations, and identification of failure points. This multicentre study aims to develop an accurate CTPA lung segmentation model, with evaluation of outputs in two diverse patient cohorts with pulmonary hypertension (PH) and interstitial lung disease (ILD).

**Methods:**

This retrospective study develops an nnU-Net-based segmentation model using data from two specialist centres (UK and USA). Model was trained (*n* = 37), tested (*n* = 12), and clinically evaluated (*n* = 176) on a diverse ‘real-world’ cohort of 225 PH patients with volumetric CTPAs. Dice score coefficient (DSC) and normalised surface distance (NSD) were used for testing. Clinical evaluation of outputs was performed by two radiologists who assessed clinical significance of errors. External validation was performed on heterogenous contrast and non-contrast scans from 28 ILD patients.

**Results:**

A total of 225 PH and 28 ILD patients with diverse demographic and clinical characteristics were evaluated. Mean accuracy, DSC, and NSD scores were 0.998 (95% CI 0.9976, 0.9989), 0.990 (0.9840, 0.9962), and 0.983 (0.9686, 0.9972) respectively. There were no segmentation failures. On radiological review, 82% and 71% of internal and external cases respectively had no errors. Eighteen percent and 25% respectively had clinically insignificant errors. Peripheral atelectasis and consolidation were common causes for suboptimal segmentation. One external case (0.5%) with patulous oesophagus had a clinically significant error.

**Conclusion:**

State-of-the-art CTPA lung segmentation model provides accurate outputs with minimal clinical errors on evaluation across two diverse cohorts with PH and ILD.

**Clinical relevance:**

Clinical translation of artificial intelligence models requires radiological review and understanding of model limitations. This study develops an externally validated state-of-the-art model with robust radiological review. Intended clinical use is in techniques such as lung volume or parenchymal disease quantification.

**Key Points:**

• *Accurate, externally validated CT pulmonary angiography (CTPA) lung segmentation model tested in two large heterogeneous clinical cohorts (pulmonary hypertension and interstitial lung disease).*

• *No segmentation failures and robust review of model outputs by radiologists found 1 (0.5%) clinically significant segmentation error.*

•* Intended clinical use of this model is a necessary step in techniques such as lung volume, parenchymal disease quantification, or pulmonary vessel analysis.*

**Graphical Abstract:**

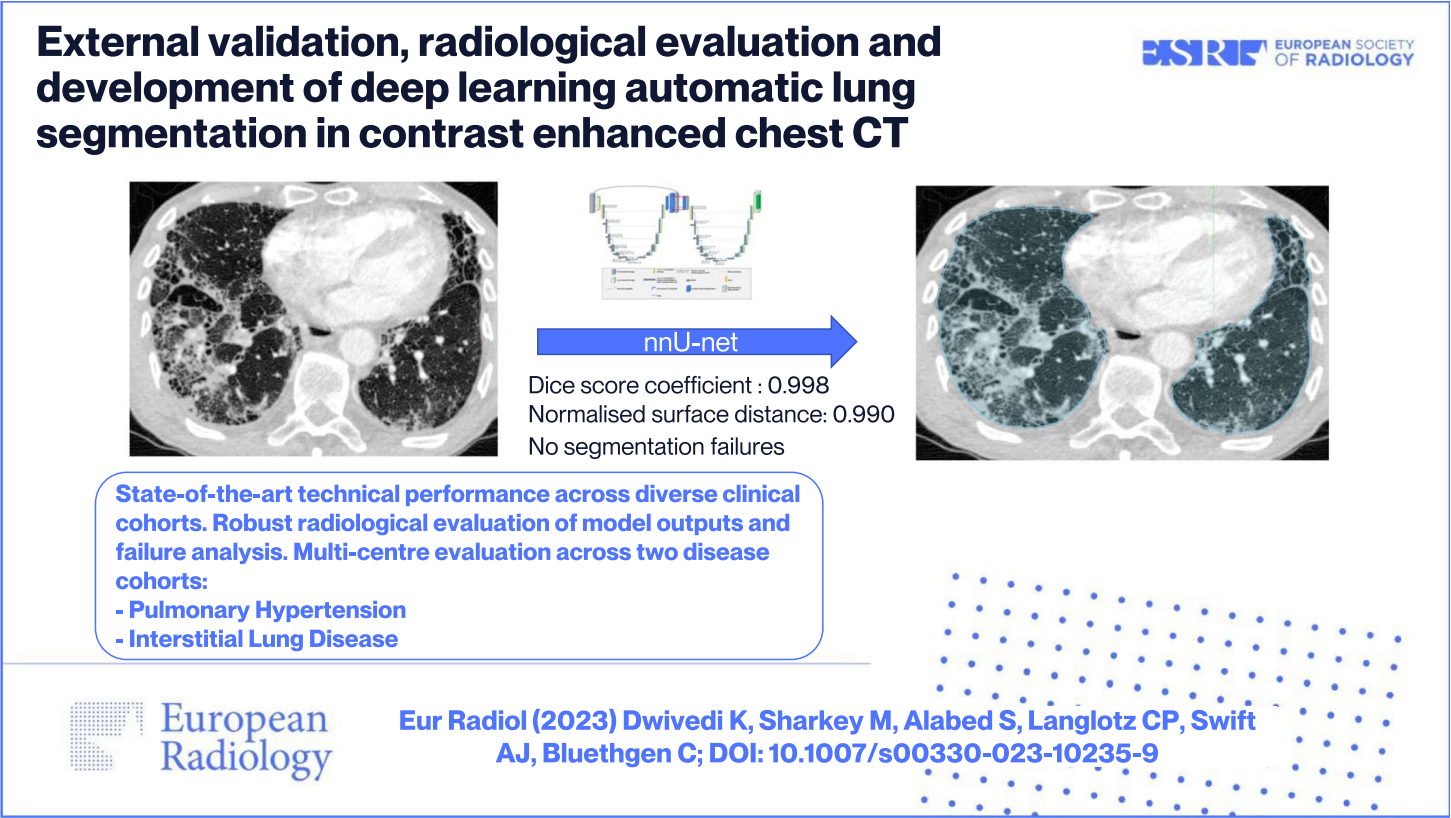

**Supplementary Information:**

The online version contains supplementary material available at 10.1007/s00330-023-10235-9.

## Introduction

Lung segmentation in computed tomography (CT) imaging is the detection and extraction of the anatomical lung boundary on each slice of the study. Automated lung segmentation is an important and necessary step in almost all chest CT clinical artificial intelligence (AI) applications such as lung nodule detection or lung parenchymal disease severity quantification. Accurate segmentation allows for detection of the region of interest (lung parenchyma) and removal of confounders (cardiac and mediastinal structures) and is important as any segmentation error propagates throughout the rest of the data analysis pipeline. Manual segmentation is time-consuming, arduous, and has significant inter- and intraobserver variability [1, 2]. It is impractical for radiologists to perform segmentation during routine clinical reading.

Within the domain of lung segmentation, a vast range of techniques exist for non-contrast chest CT, from traditional computer vision methods approaches to newer machine and deep learning approaches using convolutional neural networks, which have surpassed the performance of older methods [1, 3–6]. However, the vast majority of studies in this domain focus on technical developments with advances in underlying network architectures or processing techniques [3]. Computed tomography pulmonary angiography (CTPA) involves administration of intravenous contrast which enables assessment of the pulmonary vasculature in addition to other structures on chest CT. It is performed routinely for patients with suspected pulmonary embolism (PE) and pulmonary hypertension (PH). A known limitation of current deep learning segmentation algorithms is failure to segment high-density objects such as subpleural consolidation [3, 7, 8]. There are significant differences in the attenuation of the lung parenchyma between CTPA and non-contrast imaging due to parenchymal uptake of contrast. To the best of our knowledge, no study has trained a lung segmentation algorithm in PH or CTPA imaging and externally tested it in a heterogeneous mixed cohort of chest CT protocols.

This study develops a 3D deep learning CTPA lung segmentation algorithm using the state-of-the-art nnU-Net method [9]. The study aims are to:

1. To develop a novel state-of-the-art nnU-Net-based 3D lung segmentation model in CTPA imaging.
2. To clinically evaluate and score segmentation outputs by review from expert subspeciality thoracic radiologists, then perform failure analysis on cases with suboptimal performance.
3. To deploy and externally validate the algorithm in a heterogeneous patient cohort at another centre.

## Materials and methods

This retrospective study uses data from two patient cohorts—Sheffield, UK, and Stanford, USA. All patient data was de-identified as per GDPR and HIPAA-compliant local guidelines. Ethical approval was granted by the Institutional Review Board at both centres and approved by the UK National Research Ethics Service (16/YH/0352).

### Study cohorts

#### Sheffield (reference dataset)

Patients were selected from the ASPIRE registry, the details of which have been previously reported [10, 11]. The registry prospectively includes comprehensive clinical and radiological data on patients referred with suspected pulmonary hypertension (PH) to a tertiary referral centre. A total of 225 patients with a heterogeneous mix of normal and abnormal chest CT findings formed the study cohort. All scans were performed on two General Electric (GE) scanners with the patient in a supine position. Scanning parameters included multiple doses and kernels. All patients had thin-slice volumetric scans with contrast in the CTPA protocol. Patients had a diagnosis of either idiopathic pulmonary arterial hypertension (IPAH) or pulmonary hypertension secondary to chronic lung disease (PH-CLD).

#### Stanford (external dataset)

To evaluate the model’s generalisation performance on external data, an additional test set of CT scans was selected from a mixed cohort of patients evaluated for interstitial lung disease at Stanford Hospital and Clinics in a tertiary care setting. From a total of 2300 CT scans from 1330 patients, 28 scans were randomly selected. Scans were acquired using Siemens (*n* = 21), GE (*n* = 6), or Toshiba CT scanners (*n* = 1) and reconstructed with a variety of convolutional kernels. All scans were performed with the patient in a supine position. Twenty-five (89.3%) CT scans were non-enhanced, two (7.1%) of the scans were obtained using a CTPA protocol, and one scan (3.5%) was obtained using a CTA protocol. The underlying ILD diagnostic groups were connective tissue disease-related ILD (11 (39.3%)), exposure-related ILD (7 (25%)), idiopathic interstitial pneumonia (6 (21.4%)), post-infectious scarring of the lung parenchyma (1 (3.5%)), and IPAH (1 (3.5%)). No signs of ILD were seen in two scans (7.1%).

**Fig. 1:**
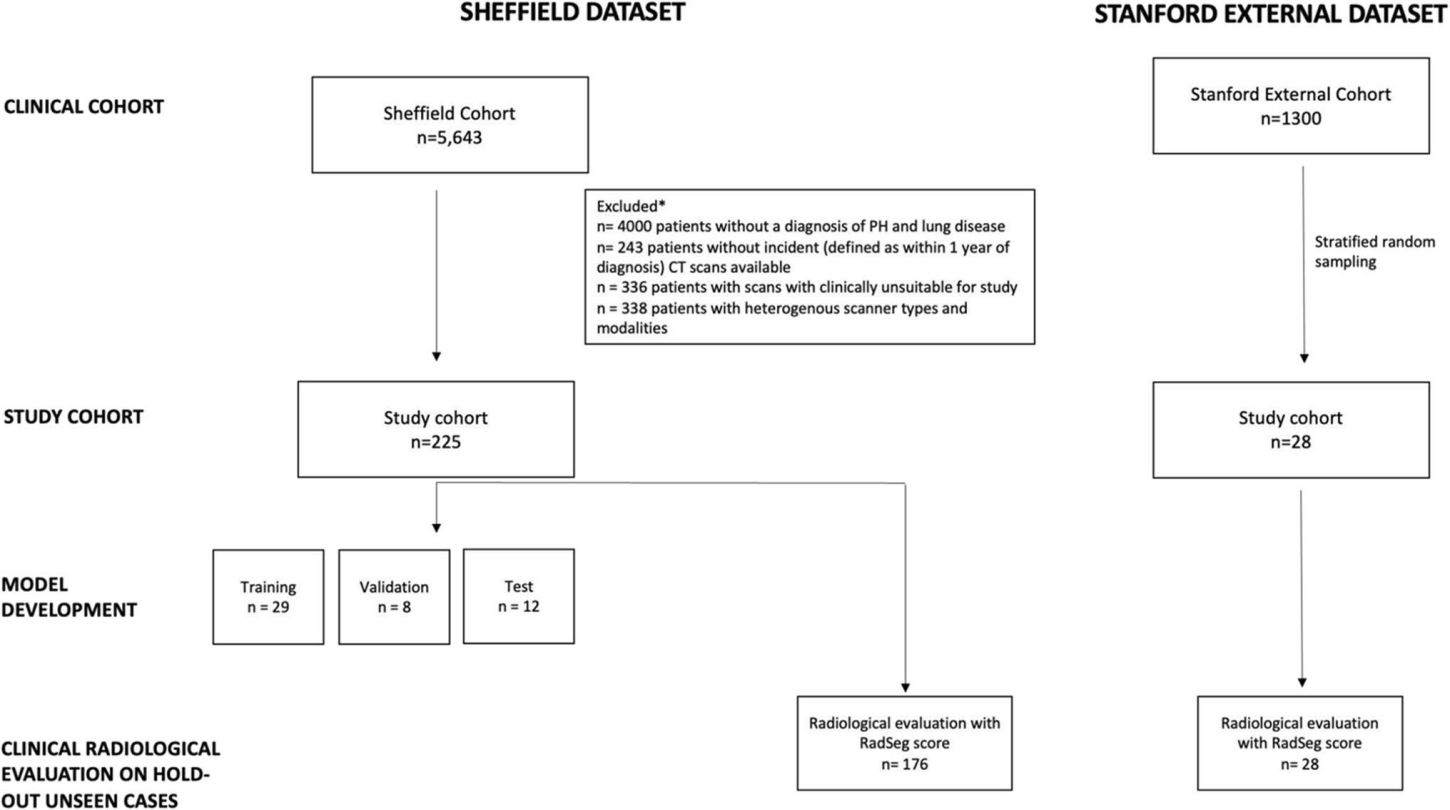
STROBE diagram showing patient selection in both internal reference datasets (Sheffield, ASPIRE cohort) and external datasets (Stanford ILD cohort). Patient groups per study stage (model development and clinical segmentation scoring) are also shown

A STROBE flow diagram for the Sheffield and Stanford cohorts is presented in Fig. 1.

## Model development

### Dataset structure

The dataset is structured at the patient level, with each patient having a unique single corresponding CT scan. The Sheffield dataset (*n* = 225) was divided into a model development (*n* = 49) and a radiological evaluation (*n* = 176) dataset. This was done to separate cases in which clinically the radiological performance is evaluated from those used for technical development. The model development dataset is further divided into training (*n* = 29), validation (*n* = 8), and testing (*n* = 12) datasets. ‘Training’ is defined as data on which the model is initially fit. ‘Validation’ is unbiased testing of the initial model fit with regularisation and early stopping to prevent overfitting. The ‘test’ dataset is an unseen unbiased dataset on which the final model is evaluated. The training cohort sample size was based on recent studies in which similarly sized cohorts utilising 3D segmentation approaches demonstrated high technical performance [3, 13]. In the internal dataset, the CT scans were volumetric acquisitions with a mean pixel size of 0.75 × 0.75 × 0.625 mm. Images were acquired with a 512 × 512 matrix with the number of slices ranging from 389 to 548. The model was tested in an external heterogenous dataset.

### Manual ground truth segmentation

MIM (MIM Software) was used to label and generate ground truth lung segmentation masks for all 49 cases in the model development cohort. A workflow was developed which used built-in operators to create an initial mask, and each step was continually reviewed by a certified radiologist. The RegionGrow tool was used to segment the lungs and airways from the trachea, manually checking for errors. Thresholds were − 350 to − 4000 Hounsfield units (HU). Tendril diameter was 4.0, with ‘fill holes’ set to none and smoothing enabled. The workflow was continuously manually evaluated, with appropriate technical parameters adjusted to improve performance. After achieving this baseline result, the scan was then manually adjusted and contoured on a slice-by-slice level by the radiologist to ensure correct segmentation of the lung border.

### Automated CTPA segmentation model

The state-of-the-art nnU-Net method is used for CTPA lung segmentation. nnU-Net is a landmark open-source deep learning–based segmentation method, which automatically configures the preprocessing, network architecture, training, and post-processing for a given task [9]. A major advantage of using nnU-Net is its open-source architecture, which is adaptable and generalisable unlike traditional highly task-focused U-Nets which can struggle with generalisability [9, 12].

**Fig. 2:**
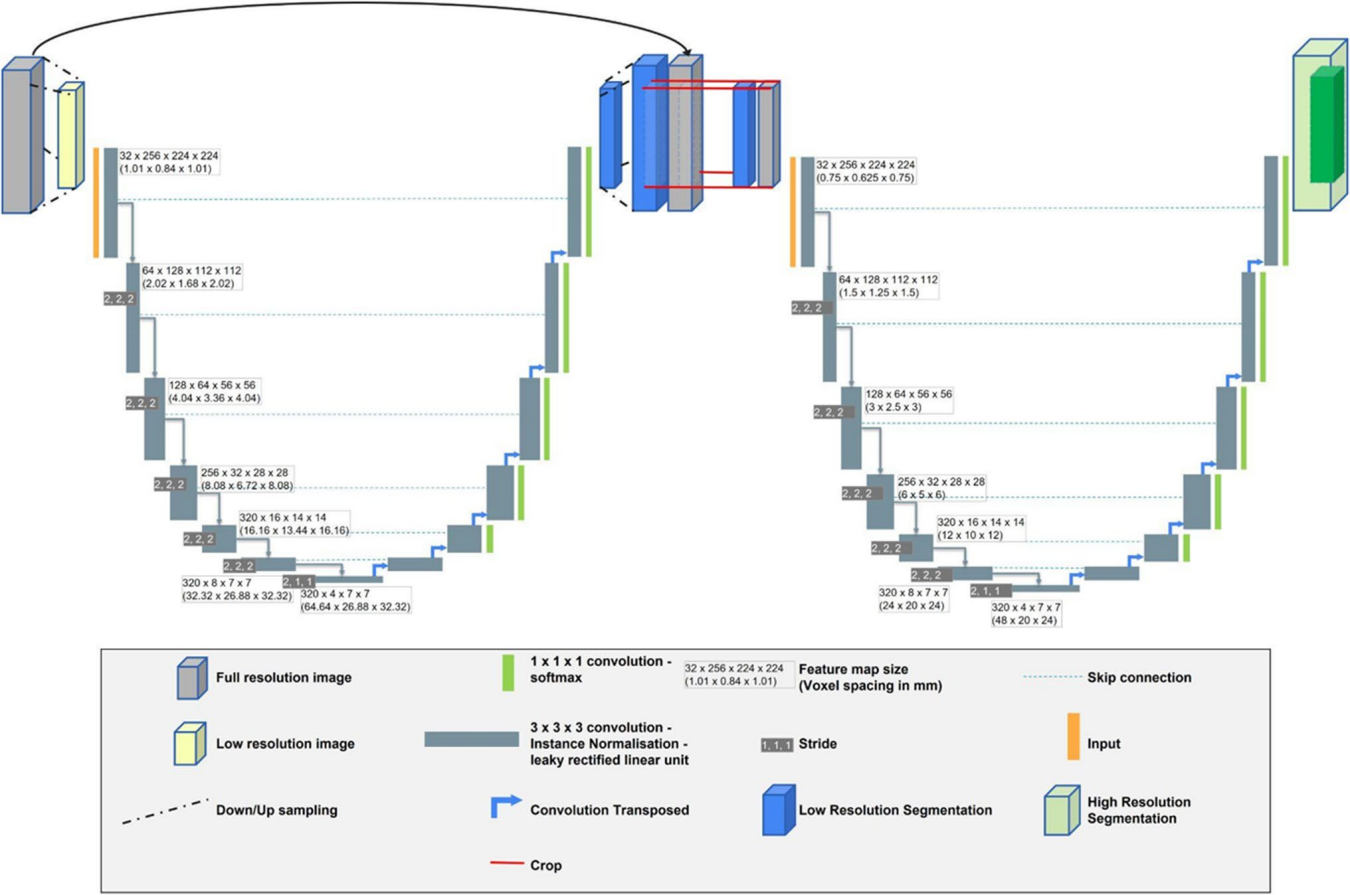
Architecture diagram. nnU-Net Architecture showing 3D U-Net with two-stage cascade used for segmentation

Preprocessing steps included truncating the HU range to − 1024 to 2500 then normalisation using SD (199 HU) and mean (− 761 HU) of the segmented lung region across all training images. A two-stage cascade 3D nnU-Net has been trained on volumetric CT data to enable 3D segmentation at close to the native resolution and to allow the model to learn context about the 3D shape of the lungs. A single-fold training approach was used, consisting of 1000 epochs with 250 mini-batches per epoch. Data augmentation during training was used with random rotation (− 30° to 30° about 3 axes, *p* = 0.2), scaling (0.7 to 1.4, *p* = 0.2), gamma correction (0.7 to 1.5, *p* = 0.3), and mirroring about 3 axes (*p* = 1). A two-stage cascade was used where the output segmentation from the first stage was passed to the second stage. During training, the output from the first stage has augmentations to randomly remove connected pixels and conduct morphological operations to enlarge/shrink the output to reduce co-adaptation. For the first stage, the training images are resampled to a mean pixel size of 1.01 × 1.01 × 0.84 mm and a 380 × 380 × 346 matrix, with a mean pixel size of 0.75 × 0.75 × 0.625 and 512 × 512 × 467 matrix for the second stage. A patch size of 256 × 224 × 224 with a batch size of 2 was used with a learning rate of 1 × 10^−3^. A diagram of the nnU-Net architecture utilised is presented in Fig. 2. Dice’s similarity coefficient (DSC), accuracy, and normalised surface distance (NSD) were calculated by comparing manual to deep learning segmentations for each case in the test cohort. Hardware used was a NVIDIA A6000 48 GB GPU, 32 Core 64 thread processor, 256 GB RAM. Post-processing steps involved the removal of regions < 250 ml in volume.

### Clinical segmentation scoring system: RadSeg

Segmentation outputs were clinically reviewed by two radiologists (K.D. and C.B.) with 4 and 7 years of experience in interpreting thoracic CT scans. An ordinal score (RadSeg score) was given to each segmentation output, scored as:

- 0—failed to output a segmentation.
- 1—lung segmented with clinically significant error.
- 2—lung segmented with minor clinically insignificant error.
- 3—full lung segmented without any clinically significant errors.

One radiologist reviewed all 176 cases from the Sheffield cohort. The 28 Stanford external cases were reviewed by two radiologists. Cases with differing scores were reviewed together and a consensus RadSeg score was given.

### Statistical analysis

All analyses from Sheffield and Stanford were performed separately; the data were not combined. Analyses were performed using R software major version 4. Categorical data are presented as number and percentage and continuous data as median and interquartile range. Segmentations were compared using an overlap-based metric (the Dice similarity coefficient (DSC)), and a boundary-based metric (normalised surface distance (NSD). For NSD, a threshold of 1.5 mm was used, as it was felt to be a clinically appropriate threshold. A *p* value of 0.05 or less was considered statistically significant.

### Role of the funding source

The funders of the study had no role in study design, data collection, data analysis, data interpretation, or writing of the report.

## Results

Patient clinical characteristics of both cohorts are shown in Table 1. Patients had a diverse range of hemodynamic, spirometric, and demographic factors.

**Table 1:** Patient characteristics for both the Sheffield and Stanford external cohorts

Characteristic	Sheffield ASPIRE PH cohort, *N* = 225				Stanford ILD cohort (external validation), *N* = 28
	Training, *N* = 29	Validation, *N* = 8	Test, *N* = 12	Radiological evaluation, *N* = 176	Radiological evaluation, *N* = 28
Demographics					
Age at scan	61 (55, 71)	70 (66, 74)	64 (57, 70)	68 (61, 75)	62 (51, 74)
Female gender	13 (45%)	2 (25%)	5 (42%)	86 (49%)	22 (79%)
Body mass index	28.2 (24.1, 34.2)	24.9 (22.8, 26.8)	27.1 (25.6, 28.7)	27.1 (23.9, 31.9)	6 (21%)
WHO functional class					
2	6 (21%)	2 (25%)	2 (17%)	20 (11%)	N/A
3	19 (68%)	5 (62%)	7 (58%)	95 (54%)	N/A
4	3 (11%)	1 (12%)	3 (25%)	60 (34%)	N/A
Smoker					
Never	9 (56%)	0 (0%)	2 (50%)	15 (27%)	15 (54%)
Ever	7 (44%)	1 (100%)	2 (50%)	40 (73%)	13 (46%)
Pulmonary function tests					
FVC % predicted	90 (78, 107)	88 (76, 93)	68 (57, 89)	83 (67, 107)	64 (52, 76)
FEV1% predicted	86 (51, 93)	66 (52, 77)	56 (37, 70)	70 (52, 87)	68 (52, 81)
FEV1/FVC Ratio	71 (61, 76)	54 (52, 75)	61 (51, 69)	68 (53, 76)	79 (75, 84)
DLCO % predicted	42 (31, 64)	31 (29, 45)	33 (28, 56)	27 (19, 42)	63 (58, 68)
Right heart catheterisation pulmonary haemodynamics					
Mean right atrial pressure (mmHg)	10.0 (6.0, 16.0)	8.0 (6.8, 10.0)	7.0 (5.0, 10.5)	9.0 (6.0, 14.0)	N/A
Mean pulmonary arterial pressure (mPAP)	44 (39, 52)	40 (32, 51)	38 (28, 48)	45 (36, 54)	N/A
Pulmonary arterial wedge pressure (mmHg)	12.0 (9.5, 15.0)	12.0 (10.0, 13.0)	10.5 (9.2, 11.8)	11.0 (9.0, 14.0)	N/A
Cardiac index (l/min × m^-2)	2.80 (2.45, 3.30)	2.74 (2.40, 3.14)	3.17 (2.58, 3.40)	2.50 (2.00, 3.25)	N/A
Pulmonary vascular resistance (wood units)	6.2 (3.9, 7.7)	6.2 (3.7, 8.9)	4.1 (3.1, 9.4)	7.3 (4.7, 10.8)	N/A
Mixed venous oxygen saturation (SvO2) %	66 (61, 70)	64 (55, 68)	71 (67, 74)	64 (58, 69)	N/A

Abbreviations used: *WHO* World Health Organisation, *PFT* pulmonary function test, *FEV1* forced expiratory volume in 1 s, *FVC* forced vital capacity, *DLco* diffusing capacity of carbon monoxide, *RHC* right heart catheterisation

### Technical results

Mean accuracy, DSC score, and NSD across the internal validation cases were 0.998 (95% CI 0.9976 to 0.9989), 0.990 (95% CI 0.9840 to 0.9962), and 0.983 (95% CI 0.9686 to 0.9972) respectively. Scores for each patient are shown in Appendix Table 1a.

### Clinical segmentation scores

#### Sheffield internal validation

There were no failures (RadSeg score 0) and all cases were successfully segmented by the algorithm. One hundred forty-six (82%) cases had a full lung segmentation without any clinically significant error (RadSeg score 3) and 31 (18%) had a minor clinically insignificant error (RadSeg score 2). No cases were segmented with a clinically significant error (RadSeg score 1). The most common reasons for segmentation errors were consolidation (9, 29%), atelectasis (7, 23%) and pleural effusion (6, 19%). These findings are shown in Table 2.

**Table 2:** Clinical evaluation of radiological segmentation (RedSeg) results and failure analysis for suboptimal performance in each cohort

	Sheffield reference cohort, *N* = 176^1^	Stanford external cohort,* N* = 28^1^
RadSeg score		
0 (failed to output a segmentation)	0	0
1 (segmented with clinically significant error)	0	1 (3.6%)
2 (segmented with minor clinically insignificant error)	31 (18%)	7 (25%)
3 (segmented without any clinically significant errors)	145 (82%)	20 (71%)
Failure analysis—reasons for RadSeg score 1 or 2		
Consolidation	9 (29%)	0
Atelectasis	7 (23%)	3 (38%)
Pleural effusion	6 (19%)	2 (25%)
Bullous emphysema	3 (9.7%)	0
Apical scarring	2 (6.5%)	0
Lung mass/nodule	2 (6.5%)	0
Fibrosis	0	2 (25%)
Azygous fissure (thickened)	1 (3.2%)	0
Collapsed lobe	1 (3.2%)	0
Patulous dilated oesophagus		1 (12%)

^1^*n* (%)

#### Stanford external validation

There were no failures (RadSeg score 0) and all cases were successfully segmented by the algorithm. Twenty (71%) cases had a full lung segmentation without any clinically significant error (RadSeg score 3) and 7 (25%) had a minor clinically insignificant error (RadSeg score 2). The reasons for segmentation errors were atelectasis, pleural effusion, and basal severe fibrosis.

The one case with a clinically significant error had a patulous dilated oesophagus secondary to scleroderma, in which the gas-filled oesophagus was included within the lung segmentation.

## Discussion

This study presents a state-of-the-art 3D lung segmentation algorithm with clinical validation across two centres in distinct well-phenotyped clinical cohorts. The two large databases used in this study represent two important patient disease cohorts, where CT imaging is routinely used. The model achieved high technical precision, indicated by high DICE and NSD scores, and clinical utility, indicated by high scores on radiological review. To our knowledge, this is the first model developed specifically in CTPA imaging and in a cohort of PH patients.

The study differs significantly in study design, patient cohort, and model evaluation compared to most segmentation studies currently in the literature. The methods were specifically designed to address common limitations that make clinical use and translation challenging. These are a lack of clinical evaluation of the segmentation output from expert radiologists, lack of diverse, heterogenous real-world clinical cohorts, and external validation. This study addresses each limitation by clinically evaluating model outputs, and by developing and testing the model on two different patient cohorts from two centres.

### Importance of radiological review of segmentation outputs

The most common parameter used to report and compare segmentation performance is the Dice similarity coefficient (DSC). However, technical parameters alone are insensitive in assessing clinical utility. Almost all studies show DSC scores > 0.97, but failure analysis of cases with suboptimal performance is rarely reported [13]. DSC itself is known to be highly reliant on structure size and provides artificially high scores by ignoring missing values [13]. The structure size limitation is particularly appropriate in the clinical use of lung segmentation, where due to the large lung volume, small volume but highly clinically significant segmentation errors—such as segmenting the oesophagus or failing to segment basal ground glass change in interstitial lung disease—have minimal impact on the reported DSC score.

We therefore propose and utilise a clinical segmentation scoring system (RadSeg score), which accounts for the small volume and clinical significance of errors. Our model had no major clinically significant errors when validated against scans from the reference centre (Sheffield) and only one after deployment in an external centre (Stanford). As radiologists are the target users in mind for such AI models, their involvement in the development and clinical evaluation of such models, we believe, will help create trust in the model’s output and help increase uptake in routine clinical practice[14, 15].

### Analysis of suboptimal outputs

**Fig. 3:**
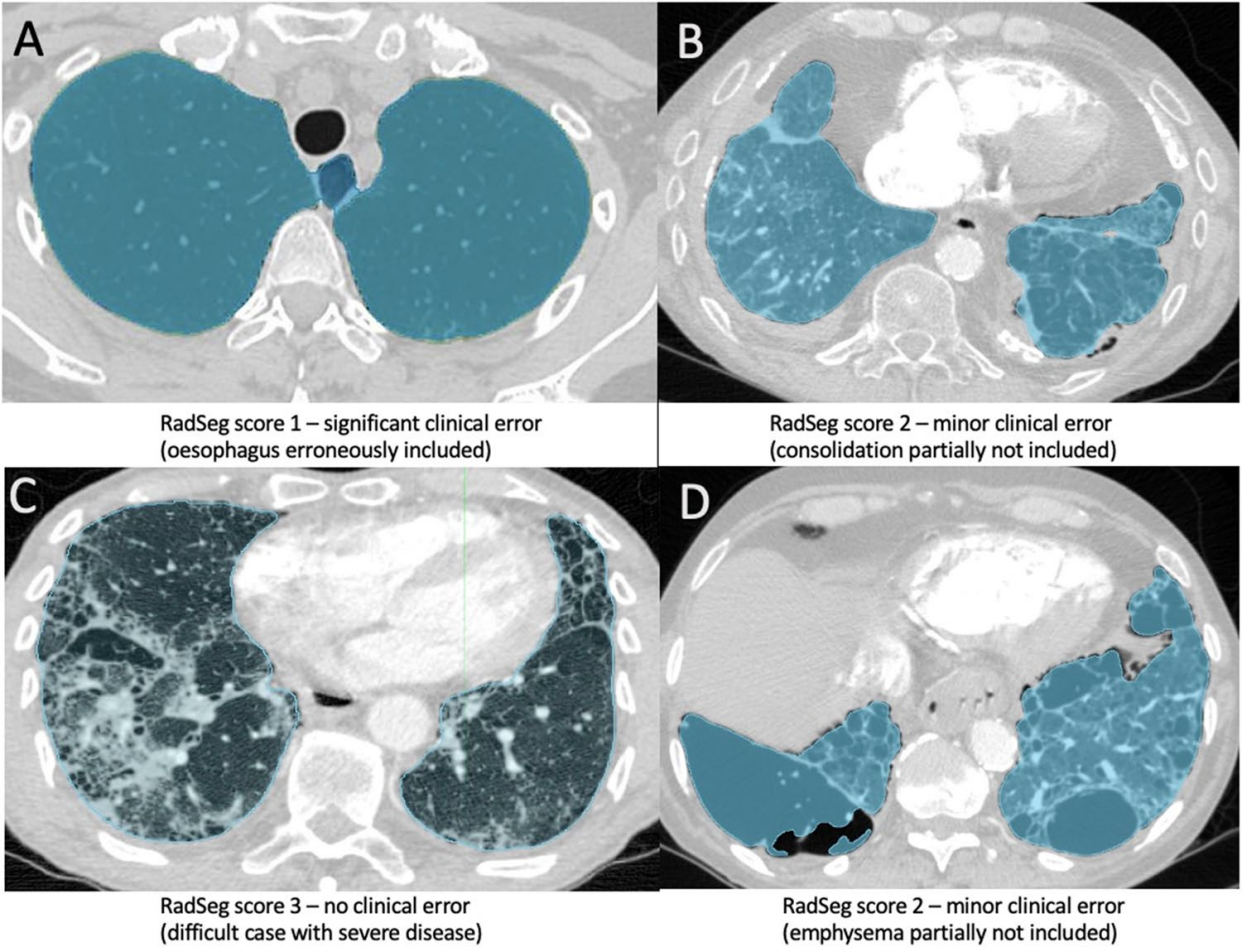
Example of RadSeg scores. Examples of cases with corresponding RadSeg scores. **A** (Score 1)—example of significant clinical error (oesophagus erroneously included). **B** (Score 2)—example of minor clinical error (consolidation at left lung base partially not included). **C** (Score 3)—example of no clinical error in a difficult case with severe lung disease. **D** (Score 2)—another example of minor clinical error (bullous emphysema partially not included)

Transparency of the algorithm limitations and causes of suboptimal performance is important to clinical trust. The training and test cohorts in our study included cases with severe parenchymal abnormalities (Fig. 3, panel C), and the majority of these accurately segmented. The most common reasons for suboptimal segmentation (RadSeg Score 1 or 2) were consolidation and atelectasis. Accurate segmentation of these high-density parenchymal abnormalities is challenging and is a limitation common to all lung segmentation models and studies[3, 7]. The causes for suboptimal performance are hard to directly compare, as they are often not reported or characterised.

Our model had no failures either in the internal or external test sets, with only one clinically significant error in the external set. External validation of any AI model is vital to test model performance and generalisability. However, only 6% of published deep learning studies in the field of diagnostics performed external validation [16]. The majority of studies with external validation demonstrate diminished algorithm performance [17]. Within the domain of lung segmentation, Yoo et al [8] found a significant drop off (DSC 99.4 in internal test to DSC 95.3 in external test) in the performance of their 3D U-Net-based model on external validation. This study performs external validation not only after deployment in a different centre, but on a different clinical cohort of patients, and demonstrates good results on clinical evaluation. The external cohort is heterogeneous both in containing patients with and without PH, but also a random mix of different volumetric chest CT protocols, with and without contrast opacification.

### Need for heterogeneous ‘real-world’ clinical cohorts

Our technical results of mean DSC of 0.990 and NSD of 0.983 compare favourably against other published 3D and 2D segmentation approaches on non-contrast scans [3, 6, 8, 18, 19]. This result likely can be attributed to the use of a state-of-the-art nnU-Net architecture combined with a heterogeneous ‘real-world’ clinical training cohort with a mix of pathology. Hofmanninger et al [3] showed that the accuracy and reliability of lung segmentation algorithms on difficult cases primarily rely on the strength of the training data, more so than the underlying model architecture. This effect is particularly pronounced on external validation against a variety of test sets, with algorithms trained on more diverse data being more generalisable. Previous studies have been limited by training on relatively homogenous cohorts, which is a by-product of utilising public datasets, which only contain normal scans or variations of a specific disease class [20, 21]. In this study, high technical performance was achieved using a relatively small training cohort size. This was influenced by similar studies in this domain, which have demonstrated excellent performance in organ segmentation tasks across modalities [9, 12, 20, 22]. nU-Net was recently used in lung ^1^H-MRI to develop a generalisable segmentation model robust to pathology and acquisition protocols and centres, but this utilised 593 scans [22]. Our study reaffirms the importance of heterogenous and well-characterised training data for the clinical translation of segmentation models.

#### Implications for practice and intended clinical use

Accurate lung segmentation is a necessity for any quantitative chest CT analysis. We envisage the clinical use of this model as the first preprocessing step in quantitative CT analysis models such as lung parenchymal disease severity quantification in PH. There is a clinical need and great interest in better characterising the severity and extent of lung parenchymal disease in PH [23]. Qualitative routine radiological report descriptions of parenchymal disease have shown to be prognostic biomarkers [24]. A new IPAH phenotype has been identified with distinct radiological and clinical characteristics [25]. These clinical scenarios will benefit greatly from automated end-to-end lung segmentation and parenchymal disease quantification models. The implications of this work also exist beyond PH, given the good performance on the external test cohort. CTPA is one of the most commonly performed investigations, mainly for the acute diagnosis of PE. Automated lung segmentation models can enable further quantitative vessel analysis and clot burden estimation [26, 27]. With appropriate transfer learning to further improve performance, this model may be used as a tool for both contrast and non-contrast imaging.

### Limitations

The training set contains only patients with a known diagnosis of pulmonary hypertension, and thin-slice volumetric CTPA protocol scans, from a single tertiary centre with a predominantly white European population. The range of lung disease in this cohort has been previously reported, and whilst this represents a realistic heterogeneous clinical cohort of patients, there is a relative skew and bias to pathological cases due to a lack of truly ‘normal’ scans [24]. A limitation to using real-world clinical data is a lack of ‘rare’ cases in the training data which can limit performance. Future work will seek to address the limitations of this study by developing and testing the DL model in a large cohort of multiethnicity patients. Despite good performance in the heterogeneous external test cohort, the intended clinical use of the developed model at this stage is limited to patients with a suspected diagnosis of PH.

In conclusion, we developed a 3D nnU-Net-based model for lung segmentation in CTPA imaging that is highly accurate, clinically evaluated, and externally tested in well-phenotyped patient cohorts with a spread of lung disease. The model is suited to important clinical scenarios of lung disease quantification in pulmonary hypertension.

### Supplementary Information

Below is the link to the electronic supplementary material.

Supplementary file1 (PDF 40 KB)

## Abbreviations

AI: Artificial intelligence
ASPIRE: Assessing the Severity of Pulmonary Hypertension In a Pulmonary Hypertension REferral Centre
CT: Computed tomography
CTPA: Computed tomography pulmonary angiography
DLCO: Diffusing capacity of carbon monoxide
DSC: Dice score coefficient
FEV1: Forced expiratory volume in 1 second
FVC: Forced vital capacity
GDPR: General Data Protection Regulation
HIPPA: Health Insurance Portability and Accountability Act
HRCT: High-resolution computed tomography
HU: Hounsfield units
ILD: Interstitial lung disease
IPAH: Idiopathic pulmonary arterial hypertension
NSD: Normalised surface distance
PAH: Pulmonary arterial hypertension
PFT: Pulmonary function test
PH: Pulmonary hypertension
PH-CLD: Pulmonary hypertension secondary to chronic lung disease
RadSeg: Radiological segmentation score
RHC: Right heart catheterisation
WHO: World Health Organisation

## Declarations

### Guarantor

The scientific guarantor of this publication is Andrew J. Swift.

### Statistics and biometry

Multiple authors have significant statistical expertise. No complex statistical methods were necessary for this paper.

### Study subjects or cohorts overlap

There may be subject overlap at the patient level with previously published works, as there have been multiple publications that have used the registry data that this study is based upon, the ASPIRE (Assessing the Severity of Pulmonary Hypertension In a Pulmonary Hypertension REferral Centre) registry.

### Methodology

• prospective

• experimental

• multicentre study
