# Supplementary material for: External validation, radiological evaluation, and development of deep learning automatic lung segmentation in contrast-enhanced chest CT
Source: Eur Radiol. 2023 Sep 29;34(4):2727–37. doi: 10.1007/s00330-023-10235-9 (PMC10957646; doi:10.1007/s00330-023-10235-9)
Supplement: Supplementary file 1 — Supplementary file1 (PDF 40 KB) [file 330_2023_10235_MOESM1_ESM.pdf]

External validation, radiological evaluation, and development of deep learning automatic lung segmentation in contrast enhanced chest CT

Electronic Supplementary Material

Table 1a. DICE and accuracy scores for each patient in performance testing.

**Case**	**Accuracy**	**Dice Similarity Coefficient (DSC)**	**Normalised Surface Distance (NSD)**
1	0.999	0.995	0.988
2	0.995	0.967	0.941
3	0.999	0.996	0.994
4	0.999	0.996	0.994
5	0.998	0.993	0.990
6	0.998	0.987	0.991
7	0.999	0.995	0.991
8	0.997	0.974	0.930
9	0.998	0.992	0.992
10	0.999	0.995	0.989
11	0.999	0.995	0.999
12	0.999	0.996	0.996
**Mean (95% Confidence Interval)**	**0.998 (95% CI 0.9976 to 0.9989)**	**0.990 (95% CI 0.9840 to 0.9962)**	**0.983 (95% CI 0.9686 to 0.9972)**

Table 1b. Scan DICOM (Digital Imaging and Communications in Medicine) information for each cohort.

**Characteristic**	**Sheffield Reference Cohort N = 225**[1]	**Stanford External Cohort N = 28**
**Scanner manufacturer**		
GE MEDICAL SYSTEMS	225 (100%)	6 (21%)
SIEMENS		21 (75%)
TOSHIBA		1 (4%)
**Scanner model name**		
LightSpeed Pro 32	64 (26%)	0 (0%)
LightSpeed VCT	161 (72%)	4 (14%)
SOMATOM Definition AS+		7 (25%)
SOMATOM Definition Edge		4 (14%)
SOMATOM Definition Flash		2 (7%)
SOMATOM Force		3 (11%)
Sensation 64		5 (18%)
Aquilion		1 (4%)
**Scan slice thickness**		
0.625	225 (100%)	1 (4%)
1.0		20 (71%)
1.25		5 (18%)
2.0		2 (7%)
**kvp**		
80	0 (0%)	1 (4%)
100	39 (17%)	4 (14%)
120	186 (83%)	23 (82%)
[1] n (%)		
